# A primer for generating and using transcriptome data and gene sets

**DOI:** 10.1242/dev.193854

**Published:** 2020-12-23

**Authors:** Chad Cockrum, Kiyomi R. Kaneshiro, Andreas Rechtsteiner, Tomoko M. Tabuchi, Susan Strome

**Affiliations:** Department of Molecular, Cell, and Developmental Biology, University of California Santa Cruz, 1156 High Street, Santa Cruz, CA 95064, USA

**Keywords:** Gene sets, Resources, Transcriptomics

## Abstract

Transcriptomic approaches have provided a growing set of powerful tools with which to study genome-wide patterns of gene expression. Rapidly evolving technologies enable analysis of transcript abundance data from particular tissues and even single cells. This Primer discusses methods that can be used to collect and profile RNAs from specific tissues or cells, process and analyze high-throughput RNA-sequencing data, and define sets of genes that accurately represent a category, such as tissue-enriched or tissue-specific gene expression.

## Abstract (precis)

**Summary:** This Primer gives an overview of the considerations required when planning and conducting transcriptome profiling experiments, from sample isolation to data analysis and display, and discusses best practice in defining gene sets for use in addressing biological questions.

## Introduction

Analysis of gene expression patterns is a common and important component of many modern biological research projects. Such analysis can provide insights into how gene expression patterns drive cell fate and function, and how mutations, drugs, diseases, physiological stimuli, and stress impact gene expression programs. The first level of analysis of gene expression patterns is quantifying levels of gene transcripts. Such ‘transcriptome’ analysis often involves determining which RNAs are characteristic of certain cells, tissues, or stages of development. This requires the availability of high-confidence lists of RNAs in those samples. Investigators face the challenges of deciding how to profile RNA populations, comparing their RNA profiles with already-published profiles and determining which transcripts are characteristic of particular cells, tissues, stages, mutants or interventions.

This Primer – intended as an overview for those new to transcriptome analysis – describes widely used methods for isolating cells and tissues and preparing samples for transcript profiling, and discusses considerations in processing RNA-sequencing (RNA-seq) data and generating lists of genes or ‘gene sets’ expressed in particular cell types. We use examples from the nematode *Caenorhabditis elegans*, but stress that the lessons and considerations extend across systems. In the last few years, single-cell RNA-seq has become increasingly popular. Because processing and analyzing such data involve many unique considerations and have been reviewed elsewhere, we do not delve deeply into single-cell RNA-seq but instead refer readers to reviews devoted to this subject for further details (Hwang et al., 2018; Luecken and Theis, 2019).

## Strategies for isolating tissues and cells for transcript profiling

**Fig. 1.**
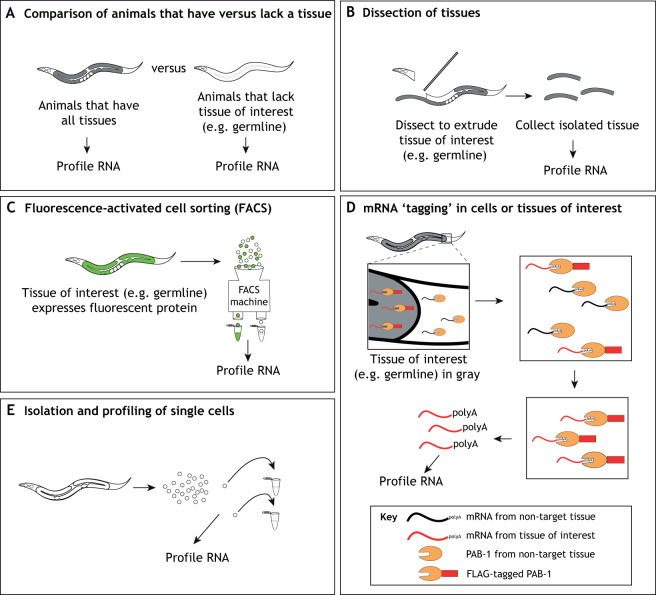
**Strategies for isolating tissues and cells for transcript profiling.** (A-E) Strategies for isolating tissues and cells for transcript profiling. Schematics representing different approaches for isolating samples for RNA profiling. Advantages and disadvantages of each method are described in the main text. See Table 1 for a reference to an example of each.

Transcriptome data from whole animals or embryos can provide important stage information, such as how a single mutation impacts an organism's transcriptome through its life cycle. Furthermore, it is typically straightforward to obtain large amounts of RNA for profiling (depending on the organism). More commonly, however, researchers are likely to want transcriptome data from particular tissues or cell types. Below, we describe approaches to isolate tissues and cell types from which RNAs can be extracted (see Fig. 1 and examples in Table 1).

**Table 1.**
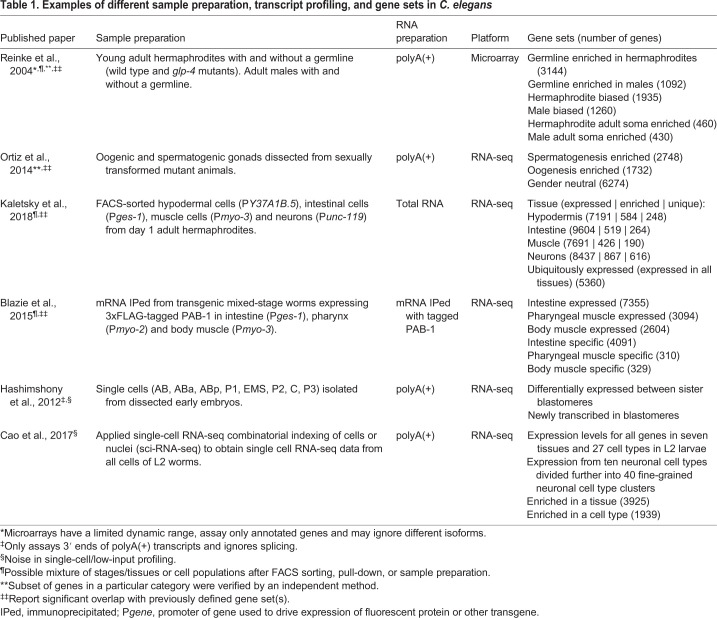
**Examples of different sample preparation, transcript profiling, and gene sets in *C. elegans***

### Comparison of animals that have versus lack a tissue or cell of interest

Comparing the transcript profiles of animals that contain (e.g. wild-type animals) versus lack a tissue or cell type of interest (e.g. mutant animals) can identify transcripts enriched in that tissue/cell type (Fig. 1A). For example, Reinke et al. identified genes that are expressed more highly in *C. elegans* germline tissue than in somatic tissues (i.e. germline enriched) by comparing levels of transcripts in wild-type adults (germline plus soma) and *glp-4* mutant animals that lack a germline (just soma) (Reinke et al., 2000, 2004). One advantage of this technique is that animals can be harvested in large quantities to yield sufficient RNAs for sensitive profiling. Although powerful, this genetic strategy assumes that all differential expression between animals is due only to the presence versus absence of the tissue/cell type, which may not always be the case; there may, for example, be compensatory changes in gene expression in other tissues. Furthermore, mutants that cleanly remove a tissue/cell type from living animals may not be available.

### Dissection of tissues

One common way to isolate tissues or cells from animals for RNA profiling is by hand dissection (Fig. 1B). This can be faster (and potentially less disruptive to the tissue) than other techniques that require several time-consuming steps, such as fluorescence-activated cell sorting (see below), but can be labor-intensive and require specialized dissection skills to collect enough material for RNA-seq (typically many thousands of cells). Some new technologies allow profiling from one or a few samples of tissue or cells, which can significantly reduce the hands-on time required to isolate enough material by dissection. Two important considerations are whether a particular tissue can be cleanly separated from surrounding tissues (for example, the germline is encased by the somatic gonad, making them difficult to separate) and the degree of cell-type complexity of the tissue of interest. Because the presence of a mixture of tissues or cell types may confound the specificity of a gene set, transcriptome data should be interpreted with caution. In some cases (particularly for small organisms or early developmental stages), hand dissection of a particular tissue may not be feasible.

### Fluorescence-activated cell sorting (FACS)

To isolate specific cell types that are difficult to isolate by dissection or to obtain sufficient quantities of cells for sensitive transcript profiling, cell sorting approaches can be used, provided the tissue of interest can be efficiently dissociated into single cells (Fig. 1C). Cell sorting by FACS requires either cell type-specific expression of a fluorescent protein (e.g. GFP) or application of a fluorescent antibody that binds to a cell type-specific cell surface marker. In either case, cells can be dissociated from large quantities of animals, after which a FACS machine separates fluorescent cells from non-fluorescent cells. One important consideration for FACS is the cell purity after sorting. Some variables that can influence cell purity are the level and specificity of fluorescent protein or surface marker expression, the stringency of the fluorescence threshold, and sorting time. FACS can take several hours, during which time cells may deteriorate, lose fluorescence intensity, and sustain alterations to gene expression (e.g. misexpression of genes involved in stress responses). It is important to ensure that the fluorescent protein (or cell surface marker) is highly expressed and specific to the cell type of interest and that cell isolation and FACS sorting time are limited. CRISPR gene-editing technology has made it straightforward to engineer cell type-specific expression of fluorescent proteins in many organisms (e.g. Dickinson and Goldstein, 2016; Paix et al., 2015; Wang et al., 2016; Dokshin et al., 2018).

### mRNA ‘tagging’ in cells or tissues of interest

Instead of profiling mRNAs from isolated cells or tissues, an alternative strategy is to ‘tag’ mRNAs in a cell type of interest and then selectively purify those tagged mRNAs (Fig. 1D). For instance, Roy et al. genetically engineered *C. elegans* to express FLAG-tagged PAB-1 [poly(A) binding protein-1] only in specific cells; mRNAs bound to FLAG::PAB-1 in those cells were then co-immunoprecipitated using anti-FLAG antibodies (Roy et al., 2002). Unlike FACS, mRNA tagging does not require dissociation of cells from tissues. Therefore, in cases for which cell dissociation is difficult, mRNA tagging may be a preferable choice. A few considerations are the need to select a promoter that will drive expression of tissue-specific FLAG::PAB-1, the assumption that FLAG::PAB-1 expression will not impact tissue health, and the need to formaldehyde cross-link transcripts to FLAG::PAB-1 to facilitate robust co-immunoprecipitation of mRNAs, which can introduce background signal. Notably, an improved mRNA tagging method called ‘PAT-seq’ [poly(A) tagging sequencing] has been developed (Blazie et al., 2015). Improvements to this method include enhancing the efficiency of FLAG immunoprecipitation by driving expression of a PAB-1 transgene with three instead of one FLAG tag and driving more reliable PAB-1 expression from a single-copy transgene inserted into the genome (instead of relying on variable expression from an extrachromosomal array).

### Isolation of single cells

In recent years, it has become feasible to profile transcripts from single cells (Hwang et al., 2018; Luecken and Theis, 2019) (Fig. 1E). A huge advantage of this strategy is that it provides cell type-specific transcriptome data. In some cases, it may be possible to identify cells of interest by size and/or shape and isolate them by hand dissection; an example is *C. elegans* early embryos (Osborne Nishimura et al., 2015). Alternatively, cells can be identified by cell type-specific expression of a fluorescent protein (as used for FACS) and then manually isolated. In some cases, researchers may instead aim to compare single-cell transcriptomes between hundreds to thousands of different cell types (e.g. to determine cell-type heterogeneity within a tumor). For such a high-throughput project, a common strategy uses microfluidics to separate a suspension of cells into thousands of individual tiny droplets, each of which will contain a single cell from the suspension and a ‘cocktail’ of ingredients to prepare an RNA-sequencing library (Xu et al., 2019). Although the dissociation of cells from animals and tissues is straightforward and inexpensive, the delivery of single cells to individual droplets and subsequent preparation of many sequencing libraries is expensive. Moreover, rare transcripts are often not detected or inconsistently detected among biological replicates (termed ‘dropouts’); consequently, transcriptome information may be incomplete and/or variable. Owing to the high variance in single-cell RNA-seq experiments, they typically require many more replicates compared with conventional RNA-seq experiments. As single-cell technologies continue to improve, such issues are likely to become less of a barrier to the use of these technologies.

## Generating and processing RNA-seq data

**Fig. 2.**
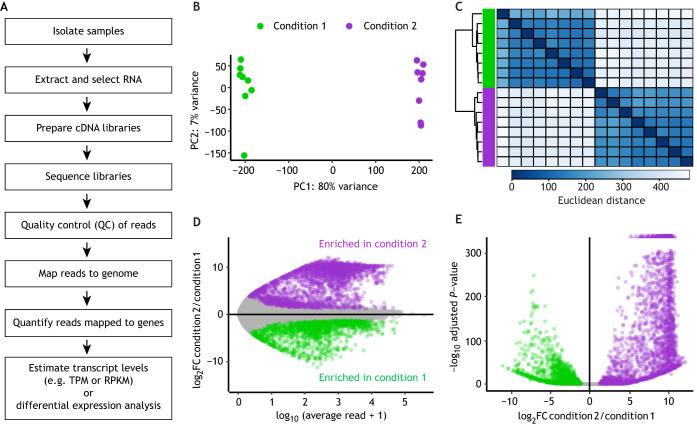
**Flow chart of steps to prepare libraries for RNA-seq and process and visualize sequencing data.** (A) Flow chart of steps. (B-E) Common ways to visualize RNA-seq data and differential expression (DE) analysis, using data from 16 libraries across two sample conditions (Ortiz et al., 2014) and the R package DESeq2 for DE analysis. Green represents condition 1, and purple represents condition 2. (B,C) Visualization of sample clustering using log-transformed counts. (B) Principal component analysis (PCA) showing distances between samples (colored dots) along the first two principal components, which together capture most of the variance between samples. (C) Heatmap showing hierarchical clustering of samples by Euclidean distance. The order of samples across columns is identical to the order down rows. Each tile represents one sample-sample comparison. The dendrogram shows the hierarchical clustering. Darker blue tiles indicate a smaller distance (larger similarity) between samples than lighter blue tiles. In both plots, samples cluster by condition, suggesting that the condition variable explains most of the variation between samples. The clean separation is a good indication of high reproducibility among biological replicates. (D,E) Visualization of DE data. Colored circles and triangles are differentially expressed protein-coding genes, defined as having at least a 2-fold difference in transcript abundance between conditions and having a *P*-value (adjusted for multiple hypothesis testing) ≤0.05. Differentially expressed genes with negative fold changes define an ‘enriched in condition 1’ gene set, and genes with positive fold changes define an ‘enriched in condition 2’ gene set. (D) MA plot showing a transcript's average abundance (read count) versus its fold change in condition 2 compared with condition 1. (E) Volcano plot showing a transcript's fold change versus its adjusted *P*-value. Triangles are genes that have a significance value that exceeds the maximum value of the *y*-axis.

After isolating RNAs from a biological sample, the next goal is to measure how many RNAs in the sample were produced from each gene (Fig. 2A). Although many studies use the term ‘gene expression’ to describe a gene's transcriptional activity, most RNA-profiling techniques measure RNA abundance, which is impacted not only by transcription but also by transcript processing and degradation. Popular genome-wide RNA-profiling techniques include high-throughput sequencing of cDNA or RNA libraries, hybridization to DNA microarrays, and serial analysis of gene expression (SAGE). These approaches are reviewed elsewhere (Schulze and Downward, 2001; Yamamoto et al., 2001; Stark et al., 2019). The current ‘go-to’ method is next-generation sequencing (NGS) of cDNA libraries (e.g. Illumina, PacBio, Ion Torrent, and Oxford Nanopore technologies). In this section, we discuss some considerations and best practices for preparing cDNA libraries for high-throughput sequencing and for analyzing and visualizing sequencing data (see Fig. 2).

### cDNA library preparation

First, researchers must decide whether to generate cDNA libraries from polyA(+)-selected mRNAs (mature transcripts with a polyA tail) or from RNA depleted of ribosomal RNAs. One key difference between these methods is the library sequencing depth needed to detect transcripts of interest, typically transcripts produced from protein-coding genes. Libraries made from polyA(+)-selected mRNA samples are highly enriched for protein-coding transcripts, and thus usually require less sequencing depth to detect transcripts of interest. Although libraries made from rRNA-depleted RNA samples should be enriched for protein-coding transcripts, they may still have a large representation of rRNA, requiring deeper sequencing to detect protein-coding RNAs. However, rRNA-depleted RNA samples do have the advantages of detecting non-polyadenylated transcripts (e.g. some histone transcripts, pre-mRNAs and non-coding RNAs) and avoiding the biases of polyA(+)-selection toward enrichment of transcripts with longer polyA tails and sequencing coverage skewed toward the 3′ end of genes.

Deciding on the appropriate sequencing depth largely depends on how much sampling of a library is needed to detect transcripts of interest. More depth is required to detect rare transcripts and when the diversity or ‘complexity’ of transcripts is high (e.g. many thousands of genes are expressed, or many transcripts are alternatively spliced). Because experimental design and goals vary widely, we recommend choosing a sequencing depth based on that reported in previous studies with a similar experimental design, or on the results of a pilot experiment. One way to identify an optimal sequencing depth is a saturation analysis, which helps identify a minimum depth needed to detect the majority of transcripts or to have sufficient statistical power for differential expression testing (Wang et al., 2012; Robinson and Storey, 2014).

Researchers must also decide how many biological replicates to prepare for sequencing. Biological replicates are cDNA libraries prepared from different collections of the same type of biological sample (e.g. the same tissue). Biological replicates are necessary to test an experiment's reproducibility, to determine biological variation and to perform statistical tests such as differential expression. It is best practice to sequence a minimum of three biological replicates of cDNA libraries; however, if high variation between replicates is expected, then more than three should be sequenced. To estimate the number of replicates needed for differential expression analysis, a power calculation can be performed using predicted values for sample variance and effect size (Conesa et al., 2016).

### Quality control of sequencing data

The first step in processing high-throughput sequencing data is quality control (QC) analysis of the raw ‘reads’. QC analysis of reads can detect technical problems that occurred during library preparation or sequencing, such as the presence of PCR artifacts or sample contamination. Some common and user-friendly tools for QC analysis are FastQC and fastp (http://www.bioinformatics.babraham.ac.uk/projects/fastqc/; Chen et al., 2018), which analyze the sequence quality and GC content of reads, the presence of adapters and duplicated reads, and more. Acceptable QC metrics depend on experimental design (for general recommendations and best practices, see Conesa et al., 2016 and Koch et al., 2018).

### Mapping of reads to a genome

To identify the genomic locus from which each read originated, reads are mapped to the organism's reference transcriptome or genome. Some popular mapping tools are STAR and HISAT2 (Dobin et al., 2013; Kim et al., 2019). For a high-quality sample, more than 70% of reads are expected to map to the genome. A low percentage of mapped reads may be a symptom of sample contamination with RNA from a different organism or excess primers or library adapters in the sequenced library. Another useful analysis for checking sample quality is visualization of read coverage over genes and exons in a genome browser (e.g. the UCSC genome browser) or metagene profile. Non-uniform coverage may reveal issues that occurred during sample and/or library preparation, such as RNA degradation or inefficient reverse transcription. Commonly used tools to perform QC of the mapping step include Picard and Qualimap (http://broadinstitute.github.io/picard/; Okonechnikov et al., 2016).

### Estimation of transcript abundance

To estimate transcript abundance, the numbers of mapped reads per transcript can be counted with tools such as HTSeq or featureCounts (Anders et al., 2015; Liao et al., 2014). Samples are then normalized to account for biases, such as sequencing depth and transcript length. The choice of normalization strategy can have a large impact on data interpretation and depends on the research goal.

If the research goal is to compare gene expression within a sample, then RPKM (reads per kilobase of transcript per million reads), FPKM (fragments per kilobase of transcript per million mapped reads) and TPM (transcripts per million) are frequently used to normalize for transcript length and library-size effects and to report transcript abundance (Mortazavi et al., 2008; Li et al., 2010). For RPKM/FPKM, the first step corrects for library size by dividing each gene's reads or fragments by the total reads/fragments in millions. The second step corrects for transcript length bias by dividing each gene's library size-adjusted counts (values after the first step) by the respective transcript length in kilobases. TPM is related to RPKM/FPKM except that the order of normalization steps is reversed. First, a gene's read or fragment count is divided by transcript length in kilobases to obtain ‘transcripts’ per gene. Then, each gene's number of transcripts is divided by the library's total transcripts in millions. An advantage of using TPM is that genes with the same read/fragment coverage will contribute equally to the total number of transcripts in the library, regardless of gene length. In contrast, with RPKM/FPKM, longer genes tend to contribute more to the total number of reads in the library. Variation in the degree of this bias can cause RPKM/FPKM values to differ between sample libraries, making it challenging to compare a gene's RPKM/FPKM value between samples. For this reason, TPM is now widely preferred over RPKM/FPKM. For a more detailed explanation and comparison of RPKM, FPKM and TPM, see https://rna-seqblog.com/rpkm-fpkm-and-tpm-clearly-explained/.

If the research goal is to compare transcript abundance between two samples, it is more appropriate to use statistical analysis of differential expression (see below), which requires other normalization methods that account for transcript composition biases (Wagner et al., 2012; Zhao et al., 2020).

After normalization, it is good practice to perform another round of QC by comparing transcript profiles of all samples in the data set, usually by clustering analyses. Principal component analysis (PCA; Fig. 2B) is a common strategy to find and visualize a few dimensions that explain most of the variance between transcript profiles (called ‘dimensionality reduction’), which helps to quickly identify transcript profiles that are similar or different (Wall et al., 2003; https://rna-seqblog.com/statquest-pca-clearly-explained/). A heatmap of hierarchical clustering (Fig. 2C) is another common visualization strategy. Biological replicates should have similar transcript profiles, and so should cluster together and away from samples that are expected to have different profiles (e.g. samples from different conditions). PCA and hierarchical clustering can also detect batch effects (differences between samples that are due to their preparation and/or sequencing in separate batches) and other technical biases (for descriptions of clustering analysis, batch effects, and best practices, see Conesa et al., 2016 and Koch et al., 2018).

### Differential expression (DE) analysis

Identifying differentially expressed genes between two conditions is a common goal in transcriptome analyses. Several widely used and free tools, such as edgeR and DESeq2, can identify and assign statistical significance to differentially expressed genes from raw count data (Robinson et al., 2010; Love et al., 2014). edgeR and DESeq2 have detailed and user-friendly explanations of the software, to help users understand and choose appropriate parameter settings for their analysis. Both packages provide methods that normalize for differences in library depth based on mapped read counts and are appropriate for comparisons between samples. Another strategy considers only reads mapped to a set of ‘negative’ or ‘control’ transcripts. For example, ‘spike-in’ transcripts added in equal amounts to all samples during library preparation are assumed to have equal abundances in the transcript profiles (Jiang et al., 2011). In a typical RNA-seq experiment, thousands of genes are separately tested for DE, creating a multiple hypothesis testing burden that increases the number of false positives: many genes that are truly not differentially expressed have low *P*-values and are incorrectly deemed differentially expressed. edgeR and DESeq2 address this problem by adjusting the *P*-value for multiple hypothesis testing. Commonly, a gene is called differentially expressed if its adjusted *P*-value (also called q-value or false discovery rate; FDR) is below 0.05 or 0.01.

Two popular ways to visualize DE analysis are MA plots (Fig. 2D) and volcano plots (Fig. 2E). An MA plot shows, for each transcript, the log fold change in transcript abundance between samples (M) versus the log of the average transcript abundance (A), giving insight into the expression levels of misregulated genes. A volcano plot shows, for each transcript, the statistical significance (usually the log of the adjusted *P*-value or FDR) versus the log fold change in transcript abundance between samples, giving insight into the statistical significance of misregulated genes.

## Gene sets

Gene sets commonly classify gene products (transcripts) as either ‘enriched/depleted’ in, ‘expressed’ in, or ‘specific’ to a particular tissue, cell type, sex, stage, or other variable. Here, we describe methods to define and assign meaning to gene sets in the context of a tissue of interest. We recommend defining gene sets using statistical criteria (e.g. *P*-values from DE analysis), but note that gene sets defined by non-statistical criteria can also yield biological insights. For cases in which non-statistical criteria are chosen, we urge researchers to consider and test the impact of those choices on the results of downstream analyses (e.g. gene set enrichment analysis).

### Defining ‘enriched/depleted’ gene sets

DE analysis is often used to classify genes as either tissue enriched or tissue depleted by identifying genes that are expressed at higher or lower levels in one sample relative to another (Fig. 2D,E). For example, a muscle-enriched gene can be defined as one that encodes a transcript found at a statistically significantly higher abundance level in a sample of muscle tissue relative to a sample of all tissues. DE analysis is the best practice to classify tissue-enriched genes, because the classification is based on statistical criteria (e.g. genes with an FDR below a specific threshold) and avoids setting arbitrary thresholds of transcript abundance (see below). As transcripts can be expressed but not enriched in a tissue, DE analysis alone usually does not define a complete tissue-expressed gene set.

### Defining ‘expressed’ gene sets

**Fig. 3.**
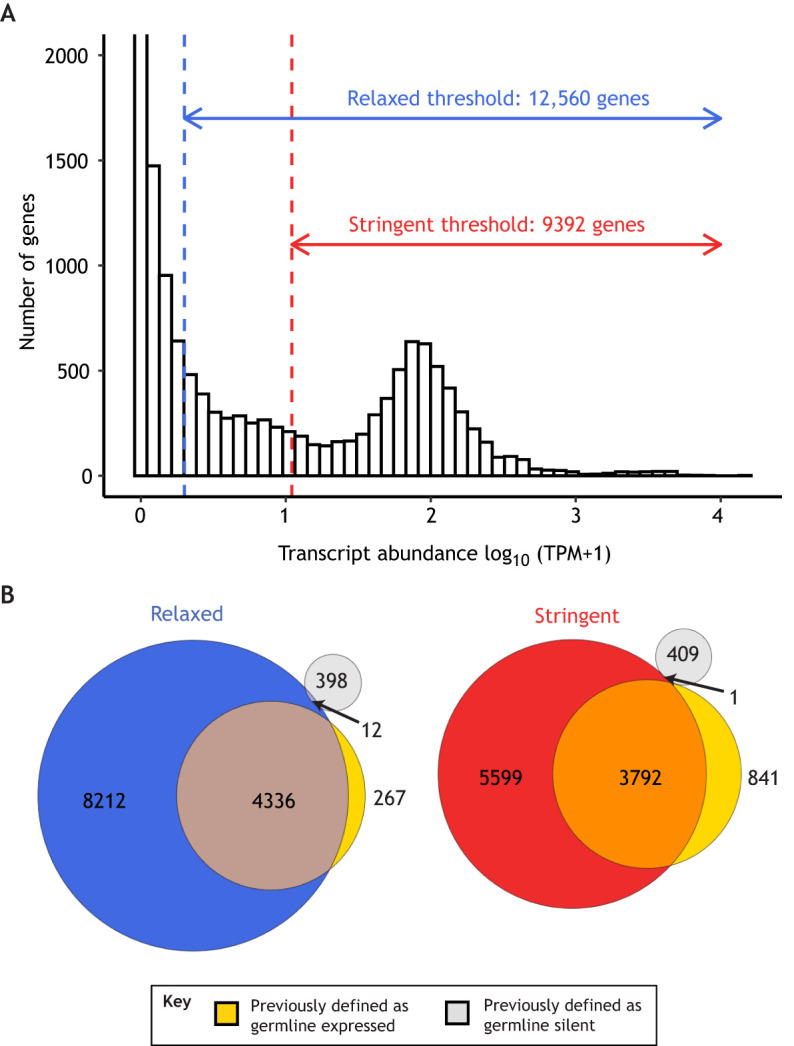
**Selecting an mRNA abundance threshold to define ‘expressed’ genes in a transcript profile.** (A) Histogram plot showing a distribution of transcript abundance values for each protein-coding gene (20,258) expressed as log_10_(TPM+a pseudo-count of 1) in a transcript profile made from germlines dissected from adult *C. elegans* (Tabuchi et al., 2018). The maximum value of the *y*-axis was artificially set to a lower value (cutting off many genes with 0 TPM) in order to show the shape of the distribution across all TPM values. The dashed vertical lines represent two choices for minimum TPM thresholds to define ‘expressed’ genes in the transcript profiles. The blue line indicates a ‘relaxed’ minimum threshold of log_10_(TPM+1) ≈0.30 (or TPM=1). The red line indicates a ‘stringent’ minimum threshold of log_10_(TPM+1) ≈1.04 (or TPM=10). (B) Venn diagrams comparing the two gene sets defined above using a relaxed threshold (left, blue circle) or stringent threshold (right, red circle) to sets of previously defined ‘germline-expressed’ genes (gold circles) and ‘germline-silent’ genes (gray circles) (Wang et al., 2009; Kolasinska-Zwierz et al., 2009). Intersections indicate genes that are found in both compared gene sets. This analysis demonstrates the trade-off of sensitivity and specificity when choosing thresholds: although the relaxed threshold is more inclusive of lowly expressed genes previously defined as germline expressed, it is also more inclusive of genes previously defined as germline silent. Despite being more inclusive, the relaxed threshold still does not include 267 genes (∼6%) previously defined as germline expressed. This may be due to differences in transcript profiling techniques used to generate the sets (RNA-seq versus SAGE) and/or the TPM threshold being too stringent.

To classify a gene as tissue expressed, researchers typically set an abundance threshold in an RNA profile. Genes that produce higher RNA levels than the threshold are called ‘expressed’, whereas those that produce lower RNA levels are called ‘not expressed’. For this strategy, the choice of threshold is usually arbitrarily set somewhere within the 1-5 TPM or RPKM/FPKM range. One way to choose a TPM threshold is by analyzing a histogram of log-transformed TPM values in a sample. Most histograms show a bimodal distribution (Fig. 3A); the mode at high TPM values reflects highly expressed genes, whereas the mode at low TPM values reflects lowly expressed genes or noise. A reasonable TPM threshold can be set to a value between these two modes. Because transcript profiles and goals differ between experiments, there is no gold-standard abundance threshold. Therefore, an appropriate threshold must be chosen and validated for each experiment, realizing that there is a trade-off between sensitivity (inclusion of genes) and specificity (inclusion of only genes that are truly expressed). Previously defined gene sets can help guide selection of a threshold (Fig. 3B). It is therefore useful to compare different thresholds: a higher-TPM threshold to generate a ‘stringent’ gene set and a lower-TPM threshold to generate a ‘relaxed’ gene set (Fig. 3A). A stringent threshold provides high specificity by omitting truly non-expressed genes, but at the cost of incorrectly excluding true lowly expressed genes (false negatives) (Fig. 3B). A relaxed threshold provides high sensitivity to detect low-abundance transcripts, but at the cost of incorrectly classifying some non-expressed genes as expressed (false positives). Because defining a gene set this way uses arbitrary criteria, we recommend evaluating its accuracy using existing gene sets and/or other types of gene expression data, such as images of fluorescent reporters or fluorescence *in situ* hybridization.

Another method is to define a ‘background’ level of TPM by measuring TPM levels in intergenic regions where no transcription occurs (Ramsköld et al., 2009; https://github.com/BgeeDB/BgeeCall). Genes that produce transcripts at a level above this background TPM value are considered to be expressed. Background TPM values can be used to calculate the FDR and a false negative rate (FNR) for the number of genes detected as expressed at different TPM thresholds. An appropriate TPM threshold can be set to the one that best balances high specificity (low FDR) and high sensitivity (low FNR) in detecting expressed genes.

### Defining ‘specific’ gene sets

A tissue-specific gene set defines genes that are exclusively expressed in one tissue compared with every other tissue. As such, they are highly desired gene sets by researchers who want to understand key genes that drive the development or function of a tissue type. To define a tissue-specific gene set, we recommend identifying transcripts that are tissue enriched by DE analysis and then identifying which of those transcripts are detected only in that tissue (e.g. by strict TPM thresholds). It is important to realize that a gene may be inappropriately classified as tissue specific if the transcript profiling data used for classification failed to detect the gene's transcripts in every other tissue. Some reasons for this might be low sequencing depth, low transcript abundance, stage-specific expression, and few cells in a tissue that express the gene (e.g. rare neuron subtypes).

### Evaluating and comparing gene sets

Researchers may want to evaluate the quality of their gene sets by comparing them with already-published gene sets. Commonly, two or more tissue-expressed gene sets that define the same tissue's transcriptome are compared to identify similarities and differences (Fig. 3B). Such tissue-expressed gene sets are expected to share a majority of genes. However, some differences in membership are expected due to differences in sample preparation, RNA-profiling technology, sequencing depth, and/or bioinformatic methods used to define gene sets. To reduce sources of variation, it is best to compare gene sets that have been processed and defined using identical methods (if possible); often, this requires one to re-process published transcriptome data. We recommend that researchers re-evaluate their gene sets as new transcriptome data become available.

### Gene set enrichment analysis

After defining a gene set of interest, a common goal is to assign biological meaning to the set using gene set enrichment analysis. This type of analysis tests whether a gene set is enriched for genes that share a common biological feature (e.g. protein function, co-regulation, signaling pathway or epigenetic environment). Therefore, it is important to use accurate and high-confidence sets of genes that define those features. Large efforts by several groups, such as the Gene Ontology Consortium, maintain and update publicly available databases of gene sets classified by biological feature (Ashburner et al., 2000; The Gene Ontology Consortium, 2019). Popular gene set enrichment analysis tools, such as GSEA, DAVID and g:Profiler, use these large databases and various statistical methods to comprehensively test many biological features for enrichment (Mootha et al., 2003; Subramanian et al., 2005; Huang et al., 2009; Raudvere et al., 2019; for a review and tutorial on best practices and various approaches to gene set enrichment analysis, see Reimand et al., 2019 and Maleki et al., 2020). Gene set enrichment analysis is an excellent method for researchers to gain insights into biology from their RNA-seq experiments and generate new and exciting hypotheses.

## Useful data sets and resources

### Some *C. elegans* transcriptome data

Table 1 presents some *C. elegans* transcriptome data and gene sets that exemplify diverse methodologies used by the community. For each study, we describe the biological samples used for transcript profiling, how they were isolated, how transcripts were profiled, and which gene sets were defined. Our goal in this Primer was to present a broad view of available data sets, not to judge or evaluate them. We recommend that researchers conduct their own evaluations of data sets they intend to use for a research goal (see the ‘Footnotes’ in Table 1 for issues to consider). An extended set of references is provided in Table S1.

### Some widely used resources

Table 2 provides links to some valuable resources that are heavily used by researchers studying human tissues and popular model organisms. WormBase, FlyBase, HumanBase and other organism-focused resource hubs collect and curate transcriptome data and provide useful tools for visualization and analysis of those data. The ENCODE and modENCODE projects produced and processed a massive amount of transcriptome data from human, mouse, *C. elegans* and *Drosophila* (Celniker et al., 2009; Hillier et al., 2009; Gerstein et al., 2010, 2014). The Expression Atlas provides transcriptome data from more than 40 species. NEXTDB is a collection of *C. elegans in situ* hybridization data. A relatively new effort, the Chan Zuckerberg Cell Atlas Initiative (https://www.czbiohub.org/projects/cell-atlas), will include transcriptome data in its ambitious goal to map every cell type in the human body.

**Table 2.**
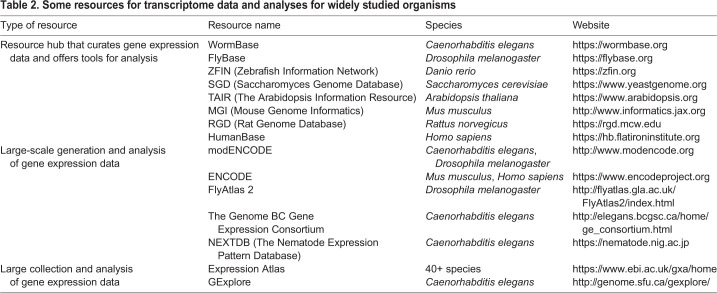
**Some resources for transcriptome data and analyses for widely studied organisms**

## Conclusions

Transcriptomic approaches are now a powerful component of biologists’ standard toolbox. A current exciting direction is defining the RNA population in individual cells in an organism, which is providing unprecedented insights into cells’ specialized gene expression patterns and functions. It is crucial that researchers new to transcriptomics learn its basics and best practices. For example, gene sets are commonly used resources, but if not defined accurately, they can lead to misinterpretation of data. Therefore, we encourage researchers to re-evaluate the reliability of gene sets as new transcriptome data and analyses become available. Our aim in this Primer was to provide a ‘launch pad’ into transcriptomics by introducing some commonly used methods and important considerations for designing transcriptomics experiments and analyzing the data. However, research goals should shape the designs and analyses. Researchers should carefully evaluate different approaches to identify which are best for their specific research goals. We hope that this Primer provides a strong foundation for scientists seeking to embrace the power of transcriptomics.
